# Exploring Sex Differences in Oxford House Residents Regarding Quality of Life, Sense of Community, and Length of Stay

**DOI:** 10.3390/healthcare13192501

**Published:** 2025-10-02

**Authors:** Daisy Diaz, Ted J. Bobak, Kelsey R. Moreno, Alexander Sikora, Leonard A. Jason

**Affiliations:** 1 Department of Psychology, Saint Xavier University, Chicago, IL 60655, USA; diaz.d09@mymail.sxu.edu (D.D.);; 2 Department of Psychology, Briar Cliff University, Sioux City, IA 51104, USA; tbobak3579@gmail.com; 3 Center for Community Research, DePaul University, Chicago, IL 60614, USA; asikora3@depaul.edu

**Keywords:** substance use disorder, sex/gender differences, recovery home, social relationships, length of stay, quality of life

## Abstract

**Background/Objectives**: Substance use disorders (SUD) pose a significant public health challenge, with 47.7 million people nationwide struggling to end their addiction. Individuals in recovery from SUDs are at an elevated risk of relapses, even after an extended period of abstinence from substances. While the importance of social relationships in addiction recovery has been extensively researched, the specific ways addiction recovery differs between sex/gender within Oxford House (OH) settings needs further research. Some evidence suggests males and females experience SUD differently and respond distinctively to recovery. **Methods**: We recruited 229 participants from 42 OH recovery homes, with 55.5% (n = 127) male participants. Moderated mediation model seven by Andrew F. Hayes was used to determine the relationship between quality of life, length of stay, sense of community, and sex/gender. **Results**: Length of stay was a significant predictor of sense of community, with longer stays associated with stronger perceived community ties. Additionally, quality of life had a robust direct effect on sense of community. We found that there is a small indirect effect of quality of life on sense of community through length of stay for females. **Conclusions**: These findings suggest that while quality of life and length of stay both independently contribute to individuals’ sense of community, the mediating role of length of stay appears to be more pronounced among females. Further research is needed to understand and address sex/gender-specific recovery experiences.

## 1. Introduction

Substance use disorders (SUDs) continue to pose a complex public health crisis in the United States, with an estimated 47.7 million individuals affected nationwide [1]. In response to the limitations of traditional treatment modalities, Oxford Houses (OH) have emerged as a community-based model of recovery. These self-governed, democratically structured residences offer individuals in recovery the opportunity to co-create sober living environments that foster mutual accountability, peer support, and long-term abstinence [2]. As decentralized alternatives to traditional care, OH reflect a broader historical shift toward empowering local, collective recovery strategies. Recovery is inherently pluralistic; individuals traverse diverse ecological, sociocultural, and psychological terrains. Therefore, recovery frameworks should remain flexible and responsive to the distinctive pathways, values, and lived experiences of those they serve.

A growing body of research underscores the centrality of social networks in shaping recovery trajectories from SUDs, with network analysis emerging as a critical methodological tool for understanding these dynamics [3]. Recovery, far from being an isolated intrapsychic process, unfolds within relational ecologies where peer affiliations, shared norms, and communal ties exert profound influence. Notably, sex-based differences in both the etiology of addiction and the responsiveness to recovery supports have been increasingly recognized [4]. Men and women may not only encounter distinct social and psychological challenges in their addiction journeys but also engage with recovery environments, such as OH, in divergent ways. Although the role of interpersonal relationships in fostering recovery is established [5], the nuanced interplay of gender, quality of life, sense of community, and length of stay within communal recovery contexts remains empirically underexplored. To address this gap, the present inquiry investigates how social relationships function within self-governed residential settings, attending specifically to the gendered contours of recovery experiences and their implications for sustained well-being and integration into sober living communities.

Quality of life (QoL) reflects a person’s subjective evaluation of their position in life, informed by cultural norms, social structures, personal values, and existential concerns [6]. The WHO’s 26-item Quality of Life Assessment—Brief Version (WHOQOL-BREF) [6] captures this multidimensional construct through four core domains: physical health, psychological well-being, social relationships, and environmental conditions [6]. Within the OH model, QoL has been identified as a pivotal variable shaping individual recovery experiences and communal dynamics. Interestingly, while demographic factors such as race/ethnicity, age, and length of stay have not demonstrated significant associations when considered alongside psychological factors like hope, QoL remains deeply interwoven with the collective functioning of the house [7]. The OH structure fosters what is termed heterophily, the formation of supportive bonds across differences in well-being levels. This principle reflects that sustainable recovery is not solely an individual achievement but a shared endeavor. A decline in one member’s QoL may reverberate across the communal network, underscoring the need for intentional, reciprocal ties that sustain both personal flourishing and communal resilience.

The Sense of Community Scale (SoC), a psychological instrument grounded in ecological theory, operationalizes the construct of community across three interdependent domains: the self (personal connection and identification), membership (interpersonal belonging and social investment), and entity (shared values and group purpose) [8]. These dimensions echo longstanding conceptualizations of community as both psychological experience and social structure. Individuals who possess a greater need for affiliation often report a stronger sense of community, suggesting that internal dispositions shape external relational bonds [9]. Furthermore, heightened house-level SoC has been linked to increased abstinence-specific self-efficacy, an individual’s confidence in maintaining sobriety in the face of temptation, highlighting the psychosocial mechanisms through which communal environments support recovery [10]. Investment in one’s community, both emotional and behavioral, further reinforces membership and strengthens overall SoC [11]. In the context of OH, this mutual reinforcement of personal engagement and collective belonging is not merely beneficial; it is essential. The sustainability of recovery within such settings hinges upon reciprocal commitment and the cultivation of shared identity, values, and responsibility.

The initial weeks of residence in a recovery home represent a vulnerable and transitional period, during which individuals face an elevated risk of early departure. This risk, however, can be substantially reduced through the cultivation of positive, supportive social relationships that foster stability and belonging [12]. A minimum stay of approximately six months in recovery homes is a threshold linked to more favorable long-term outcomes. Individuals who remain in OH for at least this duration report significantly lower levels of anxiety and alcohol consumption at one-year follow-up when compared to those in traditional recovery facilities [13], along with higher employment rates and increased income [14]. These benefits are closely associated with the individual’s capacity to form recovery-sustaining relationships, a skill that both predicts and is reinforced by extended stays [12].

The capacity to remain in recovery housing is deeply embedded within a broader psychosocial ecology. Core factors such as social embeddedness, self-efficacy, and stress management operate as the active mechanisms that shape quality of life within these communal environments [15]. Notably, longer durations of stay are associated with reductions in perceived stress and increases in abstinence-related self-efficacy, underscoring the dynamic reciprocity between psychological resilience and stable housing [15]. Yet, these individual-level processes are not isolated from structural conditions. Length of stay is also affected by contextual factors such as exposure to community violence, the racial and ethnic composition of the home, and the availability of alternative housing options. Furthermore, the presence of a significant interpersonal bond, identified as an “important person” within the house, substantially increases the likelihood of long-term residence [12]. Together, these findings point to the imperative of fostering inclusive, relationally rich environments that not only support sobriety but also affirm dignity, connection, and justice in recovery. The duration of stay in a recovery house is positively associated with higher income [15]. Improved economic stability leads to higher self-efficacy, lower stress levels, and augmented social networks, and ultimately contributes to a better quality of life. Higher wages are related to stress reduction, increased social support, and longer stays in the recovery house. Assisting new residents in finding employment significantly enhances their long-term success [15].

Emerging research underscores significant gendered divergences in both the lived experience of SUDs and the pathways to recovery. These differences are neither incidental nor solely biological but are embedded within broader sociocultural, relational, and structural ecologies. Women often enter recovery with a more complex constellation of psychosocial burdens, including elevated levels of perceived stigma, limited family or partner support, and disproportionate childcare responsibilities, factors that shape both access to and engagement with recovery settings [16]. Compounding these challenges, women tend to report lower overall quality of life in relation to hope and other psychological resilience factors when compared to men [17].

Yet women also bring distinct relational capacities to recovery, often prioritizing connection, empathy, and mutual care as foundational to their healing process. Such connections have been empirically linked to increased self-worth, interpersonal insight, and a sense of empowerment—elements crucial not only to individual flourishing but to the sustainability of community-based recovery environments [18]. In contrast, the absence of meaningful relational ties is associated with elevated depressive symptoms, diminished self-awareness, and heightened vulnerability to relapse. In OH, women’s friendships have been shown to correlate positively with self-esteem, social support, and hope, factors that, in turn, are strongly predictive of empowerment and optimism [19]. However, recovery for women can be further complicated by histories of trauma, including high rates of physical, sexual, and emotional abuse, as well as co-occurring disorders such as eating disturbances, which can impede their capacity to perceive or sustain harmonious relationships within communal living contexts [20].

The gendered nuances of substance dependence also extend to patterns of onset and use. Women tend to transition more rapidly from recreational use to dependence, often driven by affective or relational distress [16]. Conversely, men generally exhibit higher rates of polysubstance use, are more likely to experience overdose-related emergency care, and face a heightened risk of drug-related mortality [21]. Social access to substances, both legal and illicit, also skews male, with broader access often mediated by class and educational attainment [22]. Men from both lower and higher socioeconomic strata exhibit elevated risks for alcohol misuse, albeit shaped by differing structural pressures and cultural norms.

Taken together, these findings invite a more intersectional and systems-informed approach to recovery. Treatment environments must not only recognize but be explicitly responsive to the distinct psychological, relational, and structural conditions shaping men’s and women’s experiences of addiction and healing. This includes developing trauma-informed, relationally rich interventions for women, while also addressing the sociocultural conditions, such as masculine norms of emotional suppression or systemic inequalities, that may hinder men’s help-seeking and recovery trajectories. Extended stays in supportive, communal recovery settings have been found especially beneficial for women, particularly when such environments offer opportunities to build affirming relationships [23]. Ultimately, a gender-responsive recovery paradigm must move beyond individual-level treatment to embrace a broader ethics of care, interdependence, and structural transformation.

In light of the complex and gendered nature of recovery, the present study aims to deepen our understanding of how social relationships shape key recovery outcomes within OH. Given that OH are intentionally structured as single-gender recovery environments, residents may be more inclined to form social bonds and seek support from peers of the same sex, an ecological dynamic that invites closer examination. While prior research has explored the interrelations among quality of life, sense of community, and length of stay within OH, the specific influence of social relationships as mediating mechanisms remains under-investigated. To address this gap, we examine how social connectedness intersects with length of stay, perceived quality of life, and psychological sense of community among OH residents.

This inquiry is grounded in the hypothesis that both sense of community and quality of life are not only interrelated but also moderated by sex. We further posit that the length of stay in an OH will serve as a significant predictor of both individual well-being and communal belonging. By investigating these associations, we aim to illuminate how gendered patterns of relationality inform the recovery experience, particularly within self-governed, peer-supported environments. Ultimately, this work aspires to inform more nuanced and equitable interventions by highlighting the differential ways in which men and women engage with the social ecologies of recovery. Through this lens, we seek to contribute to a more just and responsive framework for sustained recovery in community-based housing models.

## 2. Method

Longitudinal data for the present study were gathered over two years, with assessments conducted every four months. The current analysis draws upon baseline data collected from OH located in three distinct states (Texas, North Carolina, and Oregon) selected intentionally to enhance the geographical and sociocultural diversity of the sample and thereby strengthen the generalizability of findings. Recruitment was facilitated by OH presidents, who introduced the study during their monthly house meetings using a standardized script provided by the research team. Following an informed introduction, professionally trained interviewers conducted individual, face-to-face sessions with residents. To safeguard confidentiality, participant responses were de-identified using numerical codes. All participants received $20 in compensation for each completed survey. The present analysis includes only those residents who participated at the baseline time point.

### 2.1. Participants 

A total of 229 individuals were recruited from 42 OH recovery residences, with males comprising 55.0% of the sample and females representing 44.5%. Participants’ mean age was 38 years (SD = 10.8). The racial composition included 82.1% identifying as Caucasian, 9.2% as African American, 1.3% as American Indian, 6.5% as Latino, and 0.4% as Alaskan Native or Pacific Islander. On average, residents had spent 10.3 months in their OH (SD = 12.6), with lengths of stay ranging broadly from one week to nearly seven years. Comprehensive survey data were gathered from all residents within each house, enabling the assignment of unique house identifiers. This approach facilitated the examination of social relationship dynamics by capturing length of stay metrics across entire communal units.

### 2.2. Statistical Analysis

Bivariate correctional analyses were conducted, and then a multiple regression mediational analysis examined the mediating effects of length of stay on the relationship between quality of life and sense of community. A moderated mediation analysis included the moderator sex of the residents. Direct and conditional indirect effects, the mediation analyses were assessed by conducting ordinary least squares regressions and bootstrapping procedures using PROCESS [24]. We evaluated an index of moderated mediation, which is a measure of differences in slopes between moderator variable categories. Effects (direct, conditional indirect effect, and the index of moderated mediation) were significant when 95% confidence interval (CI) values, based on 5000 bootstrapped resamples, did not cross zero [24].

## 3. Results

*Moderated Mediation*

**Figure 1:**
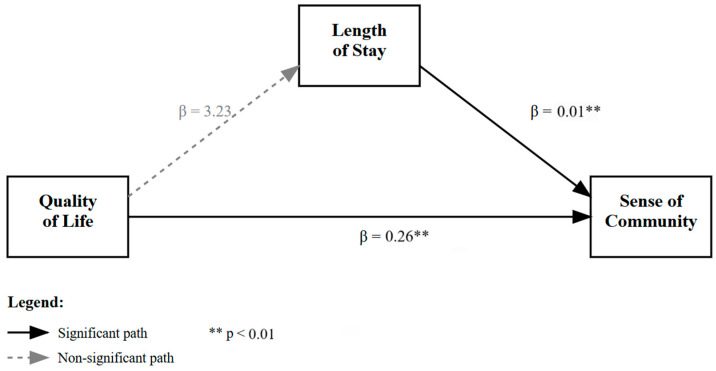
Moderated mediation effects of length of stay on the relationship between quality of life and sense of community.

**Figure 2:**
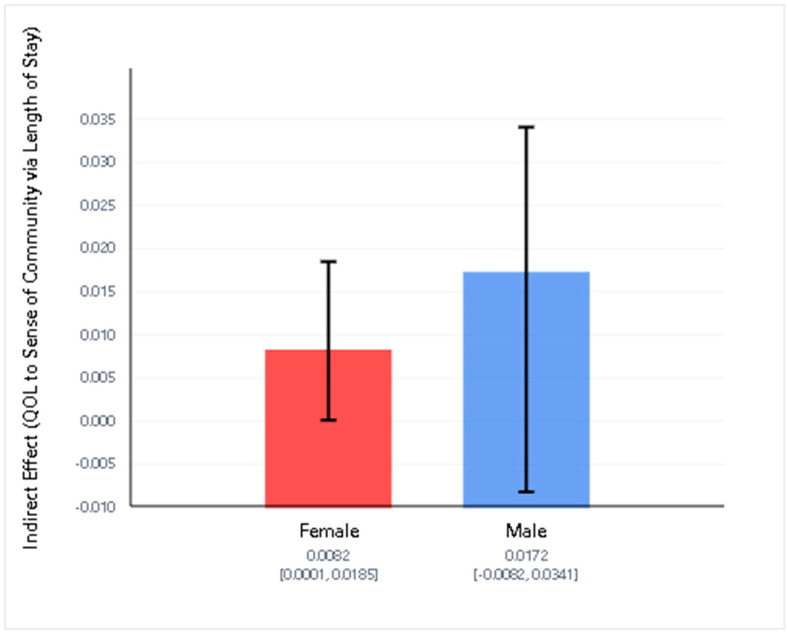
Conditional indirect effects of Quality of Life on Sense of Community via Length of Stay by gender. The indirect effect was statistically significant for females, but not for males. The index of moderated mediation was not significant. Error bars represent 95% confidence intervals.

We examined whether sex moderated the indirect effect of quality of life (QOL) on sense of community through length of stay using Model 7 of the PROCESS macro (See Figure 1 and Figure 2). The model explained a significant portion of variance in length of stay (R^2^ = 0.037, *p* = 0.035) and sense of community (R^2^ = 0.221, *p* < 0.001).

QOL was not a significant predictor of length of stay, b = 3.23, SE = 2.09, *p* = 0.124. The interaction between QOL and sex was also non-significant (b = −1.11, *p* = 0.398), suggesting that sex did not moderate the effect of QOL on length of stay. However, length of stay was a significant predictor of sense of community (b = 0.0081, SE = 0.0037, *p* = 0.027), and QOL had a significant direct effect on sense of community (b = 0.2603, *p* < 0.001).

Conditional indirect effects showed that the indirect pathway from QOL to sense of community via length of stay was significant for females (effect = 0.0082, 95% CI [0.0001, 0.0185]) but not for males (effect = 0.0172, 95% CI [−0.0082, 0.0341]). However, the index of moderated mediation was not statistically significant (index = −0.0090, 95% CI [−0.0267, 0.0201]), indicating no evidence that the mediation pathway differed significantly by sex.

These results suggest that while there is a small indirect effect of quality of life on sense of community through length of stay for females, there is insufficient evidence to conclude that this indirect effect is moderated by sex.

## 4. Discussion

The present analysis tested a moderated mediation model to examine whether the relationship between quality of life and sense of community was mediated by length of stay, and whether this indirect effect varied by sex. While the overall model accounted for a modest but statistically significant proportion of variance in both the mediator (length of stay) and the outcome (sense of community), the pattern of results only provides partial support for the hypothesized model.

Quality of life was not a significant predictor of length of stay, suggesting that individuals with higher self-reported quality of life were not more likely to remain in residence longer. Moreover, the interaction between quality of life and sex did not significantly predict length of stay, indicating that the relationship between quality of life and length of stay does not differ between males and females. These results imply that sex does not moderate the first stage of the indirect path (from quality of life to length of stay).

However, length of stay was a significant predictor of sense of community, with longer stays associated with stronger perceived community ties. Additionally, quality of life had a robust direct effect on sense of community, highlighting its central role in shaping residents’ social integration and belonging.

Importantly, although the overall moderated mediation effect was not statistically significant—meaning there is no strong evidence that the indirect effect through length of stay varied by sex—examination of the conditional indirect effects suggests that the mediating role of length of stay may be present for females but not for males. For females, there was a small but statistically significant indirect effect of quality of life on sense of community through length of stay. In contrast, this indirect effect was not significant for males. Still, since the confidence interval for the index of moderated mediation included zero, this difference between groups should be interpreted cautiously.

Taken together, these findings suggest that while quality of life and length of stay both independently contribute to individuals’ sense of community, the mediating role of length of stay appears to be more pronounced among females, albeit not to a statistically significant degree when directly comparing sexes. Future research may explore whether other factors—such as type of residence, interpersonal relationships, or gender-specific experiences—might help explain variations in how long individuals remain in a program and how this impacts their integration into the social environment in order to promote emotional well-being.

Repairing or replacing connections during active addictions becomes crucial in the recovery process, as women may turn to drugs as a substitute when dissatisfied with their relationships [18]. Previous research has highlighted women’s tendency to engage in social comparisons, which can yield negative consequences, including guilt, regret, defensiveness, dishonesty, blame-shifting, unmet cravings, and decreased happiness. Women exhibiting a stronger inclination towards engaging in negatively affected social comparisons reported lower abstinence-specific self-efficacy scores [18]. There is no direct association between higher sober housemate harmony scores and higher abstinence-specific self-efficacy scores in women.

There is a positive correlation between hope and an individual’s commitment and engagement in their OH, indicating that higher levels of engagement reflect a greater appreciation for trust and strong relationships [25]. Fostering positive friendships among women could significantly contribute to the recovery process from SUDs, as reflected by a positive correlation between such friendships and longer lengths of stay in recovery facilities [26]. Traditional masculinity norms, which emphasize self-reliance and emotional stoicism, may influence men’s help-seeking behavior and treatment engagement. Men may be less likely to seek help for substance abuse issues or to extend their stay in treatment due to concerns about appearing weak or dependent [27].

A limitation of this investigation is that a cross-sectional design limits causal inferences and interpretations on the temporal sequence of the constructs. Also, although some results are statistically significant, the effect sizes are small. Another limitation is that more than 80% of the sample consists of White participants; the lack of ethnic diversity limits the generalizability of the findings. Further studies should aim to have a more diverse sample to be more representative. Different studies can also focus on a specific racial or ethnic group (i.e., African American, American Indian, Latino, Alaskan, etc.) for any disparities among males and females that differentiate from the results with the predominantly white sample. Age and religious affiliation may also be factors that negatively or positively influence outcomes related to these results. Moreover, there is a need to investigate the impact of gender-specific recovery homes on individuals who identify as non-binary, transgender, etc. Another possible limitation in the study is that the length of stay is a more important outcome, based on its potential supporting role in the process of recovery from SUDs. This type of analysis would involve a different study, but one worth doing. 

There are practical implications of this study. To strengthen the sense of community in Oxford Houses, practitioners may consider implementing gender-sensitive strategies. For women, fostering positive friendships and addressing relational dynamics—such as reducing harmful social comparisons—could enhance engagement and prolong residence. For men, addressing traditional masculine norms that discourage help-seeking and emotional openness may improve treatment engagement and community integration.

## 5. Conclusions

In conclusion, while the overall model accounted for a modest but statistically significant proportion of variance, the findings only offer partial support for the hypothesized relationships. Further studies should investigate additional factors that may influence length of stay and sense of community, such as interpersonal relationships, residence type, and gender-specific experiences. Longitudinal designs and qualitative approaches could provide deeper insights into how emotional well-being and social belonging evolve over time in recovery settings.

## Data Availability

Data can be obtained by contacting the corresponding author.
